# Cancer Research

**Published:** 1920-05-01

**Authors:** 


### CANCER RESEARCH.

A short time ago we had opportunity to refer to the amalgamation, now officially sanctioned, between the Bland Sutton Institute of Pathology and the Cancer Investigation Department with the Medical School of the Middlesex Hospital. At a special court of governors several important and necessary recommendations dealing with the administration were adopted, and also it was agreed to make the required alterations in the standing laws of hospital, so that real effect might be given to the decisions.

The keynote of the majority of speeches made was that these efforts cannot be carried forward to their fullest possibilities without generous financial help, such as that seen in the excellent example set by Mr. S. B. Joel and Mr. J. B. Joel, who have endowed the Chair of Physics at the Middlesex with a sum of £20,000. There is no doubt that when all the scientific chairs now instituted are similarly endowed the success of the research campaign which they represent is practically assured, and there is much reason in the argument that, in the lines of the old saying "Prevention is better than cure," we should more strongly urge the public towards a practical realisation of the fact that financial support of research work will ultimately be the most useful example of philanthropic endeavour.

Nevertheless, two situations are with us at the present time, when, good as the new preventive schemes are, some considerable period must elapse before their full effect is noticeable upon the general health and physique of the community. The first is, as Lord Athlone said, that in the present state of public education in such matters, it is probably easier to obtain financial help for the actual treatment of those sick and in need than to arouse practical sympathy with plans for combating the origin of the diseases from which they suffered. The second is that there is a most pressing urgency that means of extension, endowment, and monetary aid should be found for practically all the existing hospitals, whether they be devoted to treatment, investigational work or both. Briefly, practically the whole hospital organisation of the country is in considerable financial straits, and the best hope of initial recovery lies in acting upon the principles which we have repeatedly urged in connection with the voluntary hospital system. We cannot expect our preventive medicine ideals to flourish upon an inadequately supported hospital world—and a child must be helped to walk before it can teach itself to run. We do not for one moment wish to belittle the essentially deserving nature of this research appeal, but we do wish to point out that though urgent, as are most medical reforms at the moment, it is not necessarily the most appropriate form of voluntary financial aid as applied to hospitals in the year 1920.

On the other hand, in agreement with *The Times*, we see in this type of amalgamation and University organisation a much-needed step forward in the reform of the medical training of students. The great deficiency has been a lack of reciprocity between the accepted methods of teaching and those by which the major part of useful research has been accomplished. If an example must be given, no better illustrative selection could be made than the cancer problem. All have been minutely instructed in the signs, symptoms, diagnosis and in the surgical treatment of this condition, but this process has, in exceptional cases only, provided the student with an insight into the spirit of discovery and minute observation.

From the medical teaching aspect the Middlesex scheme is magnificent to-day, and will undoubtedly produce much invaluable new knowledge in the future, but the question of immediate relative urgency of financial aid in the various phases of the medical and institutional worlds is indeed a many-sided problem.

